# Social media and functional deterioration: indicators of problematic use in university students

**DOI:** 10.3389/fpsyg.2025.1720760

**Published:** 2025-12-19

**Authors:** Myriam Carbonell-Colomer, Carlos Marchena-Giráldez, Elena Bernabéu-Brotóns

**Affiliations:** Faculty of Education and Psychology, Universidad Francisco de Vitoria, Pozuelo de Alarcón, Madrid, Spain

**Keywords:** social media, university students, addiction, gender, dependence

## Abstract

The widespread use of social media among university students has raised concerns about its potentially addictive nature and psychological impact. This study examined usage patterns, indicators of addictive behavior, and dimensions of problematic use among 526 Spanish university students (68.8% female; aged 17–25). Participants completed an *ad hoc* questionnaire and the Problematic Smartphone and Social Media Use Scales (PSSNUS). The results showed an average of 3.13 h per day spent on social media, with WhatsApp, Instagram, and TikTok being the most frequently used platforms. More than 75% of the participants reported tolerance, and 43.4% of the total participants experienced a relapse (representing 230 individuals out of the 326 who uninstalled a social media platform). Psychological dependence and loss of productivity were the most affected dimensions (especially among women). Correlational analyses revealed associations between problematic use and time spent on mobile devices, number of platforms used, and TikTok consumption. These findings identify a profile of use consistent with addictive indicators of social media use among college students and highlight the need for targeted prevention strategies and further research.

## 1 Introduction

In Spain, the number of mobile phone lines exceeds the population, with an average 1.27 devices per person (Statista, 2024; National Commission on Markets and Competition, 2025). High smartphone penetration enables constant Internet access, allowing users to communicate and access diverse information at any time. Such permanent connectivity has accelerated growth in the number of connected users and devices. However, multiple studies have shown a direct relationship between prolonged Internet use and significant functional impairment as well as various psychological symptoms (Ioannidis et al., 2019).

Access to social media has become one of the most prominent mobile phone activities. Globally, around 4.62 billion individuals, equivalent to 58.4% of the world's population, use these platforms, a number that has increased since the COVID-19 pandemic (Salas-Blas et al., 2022). In Spain, active social media usage has risen from 80% of the population in 2021 to 86% in 2024 (Nieto et al., 2022; Statista, 2024). Young adults aged 18–29 show particularly high engagement, with 88% reporting regular use, making them the demographic with the highest level of participation (Weigle and Shafi, 2023).

Excessive social media use has attracted increasing attention in clinical, scientific, and social domains (Rojas-Jara et al., 2018). A positive correlation has been noted between pathological use, fear of missing out (FoMO), and emotional dysregulation (Mascia et al., 2020). Researchers suggest that a shift from normal to problematic use may occur when individuals turn to social media as a primary (or exclusive) strategy to cope with stress, loneliness, or depression (Meynadier et al., 2025).

Part of the appeal of social networks lies in their ability to offer immediate, interactive, and dynamic communication (Di Pietro and Pantano, 2012), leading young users to report generally positive perceptions (Varchetta et al., 2023). However, ambivalence also exists. A Spanish university student participant found that while 81.6% believed social media had a positive impact, 68.55% acknowledged negative effects (Nieto et al., 2022).

University students are among the most active social media users, often considering these platforms essential to daily life (Liu et al., 2024). This demographic is especially vulnerable to excessive, potentially addictive usestemming from developmental stages involving limited self-regulation capacity (Cambra et al., 2020; Hardy et al., 2019) and frequent academic use, which weakens parental oversight and may enable overuse (Krishnamurthy and Chetlapalli, 2015). A recent systematic review reported that university students spend an average of more than 2 h daily on social media, with WhatsApp and Instagram being the most popular platforms (Carraturo et al., 2023). WhatsApp usage ranged from 1 to 5 h daily for over two-thirds of the participants, while Instagram followed similar trends.

Gender differences were also observed in usage patterns, motivations, and platform preferences. Women tend to use social media to maintain personal relationships and manage their self-image, favoring TikTok and Instagram (Pedrero-Pérez et al., 2018; Weigle and Shafi, 2023). This preference has been linked to FoMO, a phenomenon known as Fear of Missing Out (FoMO), the fear of missing out on rewarding experiences that others are experiencing (Akbari et al., 2021), and has been associated with loneliness (Piko et al., 2025), depression, anxiety (Alutaybi et al., 2020), anxiety, low self-esteem, and emotional dependence (Luque-Reca et al., 2024; Oppenheimer et al., 2024; Varchetta et al., 2023; Yin et al., 2023). In contrast, men are more likely to use social media for professional or entertainment purposes, often driven by sensation seeking and control (Jäncke, 2018; Luque-Reca et al., 2024).

The sustained increase in social media use has sparked growing concerns regarding its psychosocial consequences. Terminological ambiguity persists regarding “excessive use,” “problematic use,” and “addiction,” addiction owing to the lack of unified diagnostic criteria. Although pathological use shares symptoms with substance-related and behavioral addictions, such as dependence, tolerance, withdrawal, and relapse, it has not been formally recognized in major diagnostic manuals, such as the DSM-5 or ICD-11 (Salas-Blas et al., 2022; Meynadier et al., 2025).

This conceptual diversity has led to the development of numerous assessment tools ranging from mobile phone dependence scales (Toda et al., 2006; Kwon et al., 2013; Chóliz, 2012) to broader Internet use measures (Calvo-Francés, 2016). Recently, more integrative instruments, such as the Problematic Smartphone and Social Network Use Scale (PSSNUS; Luque-Reca et al., 2024) have emerged and validated in Spanish youth populations. This scale evaluates psychological dependence, productivity loss, preference for online interaction, desire for social recognition, and the need for control over others.

Despite the lack of formal diagnostic inclusion, robust evidence supports the negative effects of problematic social media use among young people, particularly university students. The consequences include social isolation, reduced wellbeing, distorted reality perception, anxiety, depression, low self-esteem, dissatisfaction with body image, loneliness, poor academic performance, and sleep disturbances (Escurra and Salas, 2014; Valencia-Ortiz et al., 2021; Burnell et al., 2024).

## 2 Objectives

This study aimed to describe the profile of social network use among Spanish university students, identifying consumption patterns, indicators of addictive behavior, and dimensions of problematic use.

Three specific objectives are related to this general objective:

1. Describe usage patterns
2. Identify indicators of addictive behavior (perception of use, desire to reduce use, tolerance, abstinence, and relapse).
3. Assess problematic use dimensions (dependence, productivity, social recognition, control-seeking, and preference for online interactions) and explore associated risk factors

## 3 Methods

### 3.1 Participants and design

A cross-sectional, descriptive, and correlational study was conducted using an ex post facto design. The participants included 526 young individuals, recruited via incidental non-probabilistic sampling, comprising 362 women (68.8%) and 164 men (31.2%), aged 17–25 years (M = 19.70; SD = 1.75). The sampling frame consisted of students enrolled in higher education institutions at Francisco de Vitoria University during the 2024–2025 academic year. They were approached after the classes to provide a quiet environment conducive to concentration. The sample consisted of approximately 15 classes with an average of 40 students each, resulting in a final total of about 600 participants. Of these, 526 participants completed the survey. The inclusion criteria were age between 18 and 25 years, enrollment in a higher education program, and provision of informed consent. The exclusion criteria were age outside the range and absence of social media. Incomplete responses were excluded from the analysis.

A participant size calculation was performed using a participant size calculator with a 95% confidence level and a 5% margin of error, which indicated that a minimum of 385 participants were needed to ensure adequate statistical power. Thus, the participant obtained (*N* = 526) exceeded this threshold, ensuring sufficient accuracy for the planned analyses.

### 3.2 Variables and instruments

Smartphone and Social Media Use was assessed using an *ad hoc* questionnaire collecting sociodemographic data (age, gender, and educational level), as well as phone and social media usage patterns. The questionnaire included 23 items, with numeric responses (usage time, frequency, age of initial exposure), dichotomous questions (yes/no), and multiple-choice items (e.g., most-used platforms). Addictive indicators, such as withdrawal, tolerance, and regret, were evaluated.

Problematic Smartphone and Social Media Use was measured using the Problematic Smartphone and Social Network Use Scale (PSSNUS) (Luque-Reca et al., 2024), validated in Spanish youth populations. The scale contains 18 items rated on a 7-point Likert scale (1 = strongly disagree; 7 = strongly agree) and shows high internal consistency (Cronbach's α = 0.89). It assesses five dimensions: Psychological Dependence, Productivity Loss, Preference for Online Interaction, Need for Social Recognition and Desire for Control over Others.

The indicators of addictive behavior in social media use (perception of use, desire to reduce consumption, tolerance, abstinence, and relapse) were analyzed. Users were asked about their desire to reduce the number of social networks or their time of use, as well as their perception of social network consumption and how they interact with and on them through their role (active/passive) and the repetition of behaviors seen on these platforms.

### 3.3 Procedure

The evaluation protocol was administered via the Qualtrics platform during classroom hours in a controlled silent setting. Prior to participation, the students completed an informed consent form detailing the nature of the study, confidentiality policies, and their right to withdraw at any time. These questions were addressed before the beginning of the study. Participants were also asked to verify their real-time data usage for smartphones and social media during the previous week.

Data were collected between February 1st and April 25, 2024. On average, the participants required 25 min to complete the questionnaire. No incentives or compensation was provided. The study was reviewed and approved by the Ethics Committee for Research at the Universidad Francisco de Vitoria (approval number: 41/2024), following a comprehensive evaluation of the methodological, ethical, and legal aspects of the project.

### 3.4 Data analysis

All statistical analyses were performed using the IBM SPSS Statistics version 25. Descriptive statistics (frequency, mean, standard deviation, and range) were calculated. Independent-participant *t*-tests were used to examine gender differences across variables of interest.

## 4 Results

### 4.1 Descriptive analysis of social media usage patterns

Tables 1, 2 show the descriptive data that responded to the first objective of analyzing the patterns of use of social networks among university students, identifying possible gender differences. Supplementary Table 2 in Annex 1 shows the participants and percentage of use of different social networks, also broken down by gender.

**Table 1:** Descriptive data on objective and participative mobile phone and social media usage time (in hours).

**Measure**	**Gender**	**M**	**SD**
Target time for mobile phone use	Woman	5.16^*^	2.56
	Men	4.50^*^	1.91
	Total	4.95	2.38
Target time for social media use	Woman	3.41^*^	2.14
	Men	2.45^*^	1.45
	Total	3.13	2.01
Participative time of mobile phone use	Woman	4.35	1.28
	Men	4.15	1.42
	Total	4.28	1.33
Participative time spent using social networks	Woman	3.23^*^	1.38
	Men	2.60^*^	1.27
	Total	3.03	1.38

^*^Significant differences.

^*^p < 0.05.

^**^p < 0.01.

M, Mean; SD, standard deviation.

**Table 2:** Daily hours spent on the most consumed social networks.

**Social network/ Gender**	**M**	**SD**	**Range**
WHATSAPP	1.22 (h)	1.20	[0–14.11]
Woman	1.39 (h)^*^	1.28	[0–14.11]
Men	0.93 (h)	0.87	[0–5]
INSTAGRAM	0.97 (h)	0.78	[0–5]
Woman	0.9 (h)	0.72	[0–5]
Men	1.11(h)^*^	0.88	[0–3.41]
TIKTOK	1.57(h)	1.29	[0–13.29]
Woman	1.6(h)	1.34	[0–13.29]
Men	1.3(h)	0.93	[0 – 4.70]
TWITTER	0.55(h)	0.59	[0 – 2.34]
Woman	0.41(h)	0.26	[0 – 1.22]
Men	0.73(h)	0.56	[0 – 2.34]

Significant Differences ^*^p < 0.05.

The average age of access to the first cell phone with Internet access is 12.38 years (12.26 for women, 12.65 for men). The creation of the first profile on a social network occurred at approximately 13 years of age. Gender differences were found in the age at which the first profile was created, with women creating their first profile at an earlier age (*t* = −3.001; *p* = 0.002). With an age of 12.84 years compared with 13.27 for men and 13.00 for the general population.

On the other hand, young people use an average of four social networks, which corresponds to approximately the same number of networks they have downloaded and, therefore, in which they have a profile. No statistically significant differences were found between men and women in terms of number of social networks.

In reference to the time of use (estimated by the participants vs. the time objectively recorded on the devices) (Table 1), it was found that the mean objective time of smartphone use was 4.95 h per day, of which 3.13 h were exclusively for accessing social networks, which represents 63.23% of the time spent using the mobile phone. In both cases, statistically significant differences were found, with women using smartphones (*t* = 2.97, *p* = 0.03) and social networks (*t* = 2.97, *p* < 0.001).

In terms of participants' perceptions of their own mobile phone use, significant differences were found between estimated and actual smartphone use. Participants underestimated mobile phone use by an average of 40 min per day (*t* = −7.746; *p* = 0.001), with gender differences: women tended to underestimate their mobile phone time to a greater extent, with a daily average of 49 min, compared to 21 min reported by men (*t* = −2.73; *p* = 0.006). Although no significant differences were observed between the estimated and actual time spent on social networks overall, the analysis by gender revealed a larger discrepancy for women than men, with the difference between perceived and actual time spent being more pronounced for women.

Regarding preferences in social networks, WhatsApp and Instagram are the most used social networks (downloaded by more than 94% of the survey participants). TikTok and Twitter are in third and fourth places respectively. These data can be found in more detail in Supplementary Table 2 of Appendix 1.

Significant differences were found in WhatsApp [X^2^ (2) =25.49, *p* = 0.000], Telegram [X^2^ (2) = 34.61, *p* = 0.000], and TikTok [X^2^ (2) =56.64, *p* = 0.000] in favor of greater use in girls. Boys on Twitter [X^2^ (2) = 15.20, *p* = 0.001] and Instagram [X^2^ (2) =25.29, *p* = 0.000].

In terms of daily time spent, girls spent more time connected to WhatsApp (*p* = 0.001), (*t* = 3.46), but males showed a higher daily use of Instagram (*p* = 0.025) (*t* = −2.26). It is noteworthy that the upper limit of the range of time spent on the networks is quite high: more than 14 h on WhatsApp, more than 13 h on TikTok, 5 h on Instagram, and more than 2 h on Twitter in a single day. Except for Twitter, most of the time spent online was spent by the female participants.

### 4.2 Identification and prevalence of addictive behavioral indicators

The results in Table 3 indicate that more than half of the participants (56.7%) were interested in reducing only the time spent on social media, with a greater tendency among women (58%). In general, 32.2% of the participants expressed a desire to reduce both the use and number of networks. However, the desire to reduce either the time of use or number of social networks was statistically different [X^2^ (6) = 15.14, *p* = 0.019], with this desire being more frequent among women.

**Table 3:** Perception of use and influence on their behavior.

**Measure**	**Women (*n* = 362)**	**Men (*n* = 164)**	**Total (*N* = 526)**
Reduce usage time	210 (58.0%)	88 (53.7%)	298 (56.7%)
Reduce the number of social networks	2 (0.6%)	3 (1.8%)	5 (0.9%)
Reduce both	125 (34.5%)	46 (28.0%)	171 (32.5%)
Active	165 (45.6%)	32 (19.5%)	197 (37.5%)
Passive	197 (54.4%)	132 (80.5%)	329 (62.5%)
Repetition of behavior	283 (78.2%)	87 (53.0%)	370 (70.3%)
Has not repeated behaviors	79 (21.8%)	77 (47.0%)	156 (29.7%)

Values are presented as frequency (percentage), *n* = participant size.

As for the role of our participants within the SSNs, almost 37% declared that they have an active role. In this case, significant differences were also observed [X^2^ (2) = 35.22, *p* = 0.000].

It is relevant to highlight that almost 70% of the participants repeated their behavior after seeing them in the networks. This behavior was also prevalent among women [X^2^ (2) = 34.22, *p* = 0.000], with a percentage of more than 78%.

The most repeated activity by participants, regardless of gender, was exercising (70%), followed by repeated challenges (56.70%), which was more repeated by women (66.30%) than by men (37.80%). This was followed by reading (53.50%), which was 63.50% for women and 34% for men.

The rest of the repeated behaviors were in the following order: drinking alcohol, online gambling, joining a review, watching pornography, smoking or using substances, uploading erotic photos, and self-harming. All these repeated behaviors were more frequent among boys, with the sole exception of self-harm. In the consumption of pornography and online gambling after viewing it on networks, males have three times the percentage of these behaviors as compared to females. Online criticism was twice as strong. All these behaviors, along with their percentages, can be found in Supplementary Table 5 of Appendix 1.

Tests of independence were performed to analyze the possible differences between sexes. Specifically, Pearson's chi-squared test was used for tables that met the minimum assumptions (expected frequencies ≥ 5), and Fisher's exact test was used if these criteria were not met. Statistically significant differences were found in drinking alcohol [X^2^ (2) = 15.03, *p* = 0.003], smoking or consuming other substances [X^2^ (2) =14.05 *p* = 0.010], watching pornography [X^2^ (2) = 35.50 *p* = 0.000], joining online criticism [X^2^ (2) = 8.44, *p* = 0.030], and online gambling [X^2^ (2) = 63.42 *p* = 0.000]. All of these were higher in men than in women. On the other hand, we found that women showed significant differences in the performance on challenges [X^2^ (2) = 37.55 *p* = 0.000] and reading [X^2^ (2) = 40.64 *p* = 0.000].

In addition, participants stated that 50% of respondents stated that they did not visualize these behaviors, they would not have engaged in them (15.2% of males and 53.0% of females), and 11.9% regretted repeating these behaviors.

Participants were also asked about various indicators associated with addictive behaviors on social media (Table 4). These included the desire to uninstall an application due to perceived harmful effects, the actual deletion of social networks, the presence of withdrawal symptoms (psychological discomfort after discontinuing use), relapse (return to the use of a previously deleted network), and tolerance (need to progressively increase the time of use to obtain the same effect).

**Table 4:** Frequencies of addictive indicators of social network use.

**Addictive indicators**	**Woman (*n* %)**	**Men (*n* %)**	**Total (*N*)**
Thinking about deleting the social network	307 (84.8%)	127 (77.4%)	437 (82.5%)
Delete the social network	221 (61.0%)	101 (61.6%)	326 (61.5%)
Abstinence	59 (26.7%)	14 (13.9%)	73 (13.8%)
Relapse	170 (76.9%)	59 (58.4%)	230 (43.4%)
Tolerance	280 (77.3%)	114 (69.5%)	398 (75.1%)

Values are presented as frequency (percentage). The percentage for Relapse (43.4%) is calculated based on the total participant (*N* = 526). Relapse represents 70.55% of the participants who proceeded to uninstall the social network (*N* = 326).

More than 82% of the participants considered removing the installed social network. However, only 61.51% of participants proceeded to uninstall. More than 13% of women experienced withdrawal symptoms, and psychological distress was greater in women [X^2^ (2) = 7.74, *p* = 0.021]. Of the participants who did not install the social network (*N* = 326), more than 70% (*N* = 230) confirmed having relapsed by installing the eliminated social network, with relapse being more frequent among women. [X^2^ (2) = 15.47 *p* = 0.000].

In terms of tolerance, more than 74% of the participants said that they had progressively increased their time spent using social networks(s).

The most frequently deleted social network was Instagram (34.10%) followed by TikTok (31.30%). Among other reasons, the most common were loss of time, dislike of content, and psychological discomfort (Annex 1, Supplementary Table 7). Almost half of young people (49.6%) reinstalled a deleted social network in less than a month. In addition, 17.40% reinstalled it in less than 7 days (Annex 1, Supplementary Table 8).

### 4.3 Problematic use dimensions and associated risk factors

To evaluate the different dimensions that make up the problematic use of social networks and to identify gender differences, the PSSNUS questionnaire was applied, and the results obtained for the different dimensions were analyzed (Table 5).

**Table 5:** Descriptive of PSSNUS (problematic use) and its dimensions.

**PSSNUS dimensions**	**Gender**	**M**	**SD**
PSNUSS	Woman	3.67^**^	0.88
	Men	3.32	0.90
	Total	3.56	0.90
Psychological dependency	Woman	4.25^**^	1.45
	Men	3.61	1.33
	Total	4.05	1.44
Preference online interaction	Woman	2.51	1.26
	Men	2.32	1.35
	Total	2.45	1.29
Social recognition	Woman	3.23^**^	1.57
	Men	2.66	1.42
	Total	3.05	1.54
Seeking control of others	Woman	3.02^*^	1.48
	Men	2.64	1.31
	Total	2.89	1.44
Loss of productivity	Woman	3.75	0.88
	Men	3.76	1.01
	Total	3.75	0.92

Significant differences ^*^p < 0.05; ^**^p < 0.01.

The overall mean scores on the problematic use questionnaire were higher for girls (3.67) than for boys (3.32) (*p* = 0.000) (*t* = 4.24). The highest score was given for the psychological dependence dimension (4.05), followed by the loss of productivity (3.75). Regarding gender differences in the different dimensions assessed, a higher score was found for women in the psychological dependence variable (*p* = 0.000) (*t* = 4.79), seeking social recognition (*p* = 0.000) (*t* = 4.12), and seeking control from others (*p* = 0.004) (*t* = 2.93).

To analyze whether the total score on the PSSNUS and the scores obtained on the different dimensions of the PSNUSS were significantly related to the addiction indicators evaluated, specifically abstinence, tolerance, and relapse, Student's *t*-test for independent participants was performed. The corresponding results are listed in Table 6.

**Table 6:** Relationship between PSSNUS scores and addiction indicators.

**Addiction indicators**	**PSNUSS**	**Psychological dependence**	**Online interaction preference**	**Social recognition**	**Control search**	**Loss productivity**
Abstinence Yes	4.09 (0.88)	4.85 (1.27)	2.82 (1.39)	3.94 (1.68)	3.39 (1.43)	3.89 (1.00)
No	3.38 (0.84)	3.76 (1.39)	2.23 (1.16)	2.94 (1.49)	2.70 (1.40)	3.71 (0.91)
Tolerance Yes	6.67 (0.86)	4.23 (1.39)	2.53 (1.33)	3.13 (1.52)	2.97 (1.46)	3.82 (0.88)
No	3.24 (0.93)	3.49 (1.45)	2.22 (1.13)	2.85 (1.61)	2.66 (1.34)	3.54 (1.01)
Relapse Yes	3.67 (0.88)	4.13 (1.41)	2.44 (1.31)	3.33 (1.58)	3.04 (1.47)	3.84 (0.89)
No	3.22 (0.84)	3.70 (1.45)	2.18 (1.04)	2.76 (1.53)	2.39 (1.23)	3.53 (0.99)

Values are presented as mean (standard deviation).

Statistically significant differences were observed according to the presence or absence of withdrawal symptoms, both in the overall PSNUSS score and in most dimensions, except for the loss of productivity dimension. Specifically, participants who reported experiencing withdrawal after stopping using a social network had significantly higher scores on the PSNUSS overall scale (*p* = 0.000; *t* = 6.33) as well as on the dimensions of psychological dependence (*p* = 0.000; *t* = 6.03), preference for online interaction (*p* = 0.001; *t* = 3.31), social recognition (*p* = 0.000; *t* = 4.89), and seeking control over others (*p* = 0.000; *t* = 3.70).

Similarly, statistically significant differences were found as a function of the tolerance indicator. Participants who reported experiencing a progressive increase in social network use over time scored significantly higher on the PSNUSS global score (*p* = 0.000; *t* = −4.803), as well as on the dimensions of psychological dependence (*p* = 0.000; *t* = −5.26), preference for online interaction (*p* = 0.011; *t* = −2.57), seeking control over others (*p* = 0.023; *t* = −2.28) and, in this case, also in the loss of productivity dimension (*p* = 0.003; *t* = −3.02).

Regarding the relapse indicator, significantly higher mean scores were observed in participants who claimed to have experienced at least one relapse in social network use. These differences were evident both in the overall PSNUSS score (*p* = 0.000; *t* = 4.29) and in the dimensions of psychological dependence (*p* = 0.015; *t* = 2.43), social recognition (*p* = 0.003; *t* = 2.95), seeking control over others (*p* = 0.000; *t* = 4.06) and loss of productivity (*p* = 0.006; *t* = 2.73).

Subsequently, a multivariate analysis of variance (MANOVA) was conducted to examine the effects of gender and addiction indicators (abstinence, tolerance, and relapse) on the different dimensions of the PSNUSS. Gender, abstinence, tolerance, and relapse were included as independent variables, as were the interactions between gender and each of these indicators.

The results, presented in Table 7, revealed statistically significant effects only for abstinence (*p* = 0.012) and tolerance (*p* = 0.005).

**Table 7:** Summary results of the multivariate analysis of variance (MANOVA).

**Dimension**	**Principal effect**	***F***	***df***	***P***
PSNUSS total	Gender	3.153	2	0.044
	Abstinence	11.247	1	0.001
	Tolerance	10.367	1	0.001
Psychological Dependence	Gender	5.613	2	0.004
	Abstinence	8.722	1	0.003
	Tolerance	8.443	1	0.004
Preference Online Interaction	Abstinence	5.501	1	0.020
	Tolerance	5.775	1	0.017
Social Recognition	Abstinence	9.757	1	0.002
Loss of Productivity	Tolerance	7.179	1	0.008
Control Search	Abstinence	3.825	1	0.051
Relapse	-	-	-	>0.05

F, F statistic; DF, degrees of freedom; p, probability value.

Univariate analysis by dimension showed significant differences as a function of sex (*p* = 0.004) in the PSNUSS total score. Likewise, differences were observed as a function of abstinence on the dimensions of preference for online interaction (*p* = 0.020) and social recognition (*p* = 0.002) and as a function of tolerance on the dimensions of psychological dependence (*p* = 0.004) and loss of productivity (*p* = 0.008). In contrast, the relapse indicator showed no significant effect on any of the dimensions analyzed.

Finally, *post hoc* analyses revealed that women scored significantly higher on the PSNUSS total score (*p* = 0.006), as well as on the psychological dependence (*p* = 0.001) and social recognition (*p* = 0.021) dimensions.

Finally, to answer the last objective, a Pearson correlation was made with each of the dimensions of the questionnaire, with other objective indicators such as time of consumption (both mobile and social networks), the number of social networks used, and the use of the most used social networks per hours per day. These data are shown in Table 8. In which we found moderate correlations between the PSNUSS questionnaire and time spent on both mobile and social networks, as well as the use of TikTok in particular.

**Table 8:** Pearson correlation results between PSNUSS and other relevant addictive indicators.

**Addiction indicators**	**PS**	**PL**	**PD**	**POI**	**SR**	**CS**
Mobile time	0.254^**^	0.133^**^	0.231^**^	0.123^**^	0.061	0.222^**^
Social networking time	0.204^**^	0.090^*^	0.218^**^	0.045	0.083	0.185^**^
Number of social networks used	0.143^**^	0144^**^	0.110^*^	0.070	0.026	0.092^*^
Use WhatsApp	0.069	−0.021	0.114^*^	0.034	−0.010	0.082
Use Instagram	0.092	0.042	0.081	0.089	0.027	0.032
Use TikTok	0.162^**^	0.064	0.128^*^	0.036	0.151^**^	0.111^*^
Use Twitter	0.070	0.028	−0.85	0.117	0.259^*^	−0.053

PS, PSNUSS; PL, Productivity Loss; PD, Psychological Dependence; POI, Preference Online Interaction; SR, Social Recognition; CS, Control Search.

Significant Differences ^*^p < 0.05; ^**^p < 0.01.

Productivity loss correlated positively with time spent on mobile phones and social networks, as well as the number of social networks used at the time. Indicating that the more time or social networks, the lower the productivity.

Psychological dependence was associated with both times spent on mobile phones, general social network use and specifically WhatsApp and TikTok moderately and with the general number of social networks. Showing that the more time spent in general on the mobile phone, social networks and specifically WhatsApp and Instagram, the greater the dependence.

Control seeking correlated moderately with time spent using the phone and social networks, as well as with the number of social networks and specifically with TikTok consumption. And social recognition was moderately correlated with TikTok use and Twitter use exclusively.

## 5 Discussion

The main objective of this study was to provide a detailed characterization of the use of social networks in the Spanish university population. To this end, three specific objectives were set out: to analyse usage patterns, to identify indicators of addictive behavior and to evaluate the dimensions that make up the problematic use of these platforms.

### 5.1 Objective 1: usage patterns

The use of social networks in the participant analyzed was 100%, exceeding the percentages reported in previous studies, which were around 88 % (Weigle and Shafi, 2023). The average age of starting to use these platforms was 13 years, with a slightly lower average for females (12.84 years) compared to males (13.27 years). It should be noted that, according to current legislation in Spain, Organic Law 3/2018 on the Protection of Personal Data and Guarantee of Digital Rights (BOE-A-2018-16673), the minimum legal age to create a profile on social networks is 14 years, a threshold that is not reflected in the data obtained. Early initiation of social media use has been identified as a risk factor in the development of addictive behaviors, both related to substances and behavioral addictions, such as online gaming or video games (Arias and Orio, 2024).

Regarding the number of social media platforms used, the average was four platforms per person, with no significant differences between genders. The most frequently used apps were WhatsApp, Instagram, and TikTok, present in many cases. The average daily time spent on social media was 3.13 h, within a total mobile phone usage of 4.95 h per day, representing 63.23% of total device usage time. Preference for specific social networks is consistent with previous research (Pedrero-Pérez et al., 2018; Carraturo et al., 2023), which reports widespread use of WhatsApp (99 %), followed by Instagram (over 94 %) and TikTok (over 73 %), suggesting a moderate but widespread pattern of use in this population.

Preferences in the use of social networks showed slight variations by gender. For men, the most used platforms were Instagram (1.11 h per day), TikTok (1.30 h) and WhatsApp (0.93 h), while women spent more time on TikTok (1.60 h), WhatsApp (1.39 h) and Instagram (0.90 h). No previous studies have been found that report a higher prevalence of Instagram use among men, which could represent a particularity of this participant.

The sum of time spent on these three platforms (the most used in Spain) amounted to 3.34 h per day for men and 3.98 h per day for women. This difference is consistent with recent research indicating higher screen time and social network use among women compared to men (Dorrestein et al., 2025).

On the other hand, a discrepancy was observed between perceived and actual usage time. On average, participants underestimated their mobile phone use by approximately 40 min per day, with the difference being greater for women (49 min) than for men (21 min). In terms of social network use, the perception was more accurate, although an underestimation was detected only in women, with an average of 14 min per day.

Moreover, the profile declared by the participant was generally passive, especially in men (80.5% men vs. 54.4% women), so that most of this time spent is not spent on any activity such as commenting, uploading photos or talking to other users. This passive use (consuming information without interaction) has been associated with depressive symptomatology, negative affect or envy (Tandoc et al., 2015; Verduyn et al., 2021). On the other hand, a significantly higher percentage of women showed an active role (45.6% vs. 19.5% in men). Active activities can be beneficial in certain contexts, such as social connection. However, both the creation and viewing of content (usually selfies or photographs) can have negative effects on mood and body image (Burnell et al., 2024). Dorrestein et al. (2025) stresses that content, regardless of whether it is viewed or created, is a contributing factor to body dissatisfaction and mental health problems, especially for women.

Furthermore, regardless of the role acquired within SSNRs 70% of our participant reported repeating behaviors seen on SSNRs, with a significantly higher percentage in women (78.2% vs. 53%). This may be related to various factors such as Fear of Missing Out (FoMO) or envy triggered by exposure to idealized content or luxurious experiences shared by others (Liu et al., 2024).

A recent study by Wang et al. (2019) identified that the envy variable mediates and enhances depressive symptoms in the context of social media use. The results showed a positive relationship between envy and passive use of these platforms, as well as between envy and increased depressive symptoms. However, no direct significant relationship was found between passive social network use and depression, suggesting that envy may play a key role in this link.

The behaviors most frequently replicated by young people after exposure to social networks were physical exercise (70.30%) and participation in viral challenges (56.70%), the two most common activities among both females and males. The popularity of these challenges has raised concerns about their potential negative consequences, especially among young people with increased neurological vulnerability and need for social validation (Steinberg, 2010). Some of these challenges, such as the Blackout Challenge—which involves causing loss of consciousness by choking—or the Benadryl Challenge, have been associated with serious consequences, including death (Children's Hospital of Philadelphia, 2023).

From these two main behaviors, gender differences begin to emerge. Females showed a lower diversity of repeated behaviors, with reading (66.50%) as the predominant activity, followed at a distance by drinking alcohol (11.30%) and participating in online reviews (6.90%). In contrast, males reported a greater variety of behaviors, including online gambling (25.60%), pornography use (18.30%) and alcohol consumption (14.60%), with percentages three times higher than those observed in females for these activities. It is worth noting that 11.9% of the participants reported having regretted having engaged in any of these behaviors. In addition, 50% indicated that they would not have engaged in them if they had not previously visualized them on social media, highlighting the potential impact of these platforms on young people's imitative behavior.

### 5.2 Objective 2: indicators of addictive behavior

In this regard, more than 55% of the participant expressed a desire to reduce the time they spend on these platforms, with this intention being more prevalent among females (58%) than males (53.7%).

The percentage increases exponentially when asked about the thought of uninstalling a social network, where more than 82% of our participant stated this intention, although only 61.51% carried it out. In these differences we can observe the discrepancy among young people found in Nieto et al. (2022) between the benefits and losses observed by university students, which marks a key component according to Griffiths's (2005) model of addiction: conflict.

In this model of addiction components, the author also includes salience, mood modification, tolerance, abstinence and relapse. We consider that salience is answered by its prevalence and the amount of social network use, in addition to the university students' own assertion that social networks are essential to our lives (Liu et al., 2024). It can also be answered by the indicator of tolerance, in which more than 74.3% of the participant claimed to have felt it.

The abstinence component indicated lower results, but equally worrying as more than 22% claimed to have felt this discomfort after the impossibility of using social networks (in this case women showed significant differences 26.7% compared to men 13.86%).

Relapse was expressed by more than 70.55% of those participants who had previously uninstalled the social network (N=326), again with significant differences between women 76.92% and men 58.42%. Furthermore, within these relapses (of which more than 50% occurred in less than 1 month), a percentage of more than 77% had proceeded to withdraw because their use generated unpleasant feelings, psychological discomfort or guilt, which could be indicated as an indicator of this last component of the mood modification model.

Our results thus reaffirmed the second objective, joining the latest results that speak of significant symptomatic overlap with behavioral addictions (Rodríguez et al., 2023) and that have these variables in common; tolerance, abstinence, relapse and conflict (Andreassen et al., 2016; Kuss and Griffiths, 2011).

### 5.3 Objectives 3 and 4: problematic dimensions and risk factors

The results on the PSNUSS test were 3.56 (3.67 for females and 3.32 for males). An increase was observed with respect to those obtained in the validation of the tool, where boys obtained a mean of 2.99 and women 3.23 (Luque-Reca et al., 2024).

The dimension with the highest prevalence among university students is psychological dependence (4.05) followed by loss of productivity (3.75), in third place we find social recognition (3.05), followed by the search for control (2.89) and finally the preference for online interaction (2.45). There is no difference in position according to gender, but there are significant differences in the overall mean of the PSNUSS, psychological dependence, seeking recognition and seeking control, with all the means being higher in women.

These results may provide an answer to the reasons for social network consumption, which indicate that women tend to use social networks mainly to strengthen personal ties and manage their self-image. This preference has been associated with factors such as fear of missing out (FoMO), anxiety, low self-esteem, which are related to this search for recognition, and emotional dependence (Luque-Reca et al., 2024; Oppenheimer et al., 2024; Varchetta et al., 2023; Yin et al., 2023).

In contrast, men seem to be more oriented toward a functional or professional use of networks, linked to sensation seeking (Jäncke, 2018; Luque-Reca et al., 2024). This would make sense of the diversity of behaviors imitated through social networks and the fact that all of them have a high sensory character (playing sports, watching pornography, online gambling, criticism, etc.).

However, although previous analyses have found gender differences in variables such as age of onset of social network use (Nesi and Prinstein, 2015), frequency and amount of use (Twenge and Martin, 2020), preferences for certain platforms (Muscanell and Guadagno, 2012), as well as emotional or social motives for use (Barker, 2009), these differences do not hold when analyzing the impact of indicators of addiction, such as abstinence or tolerance.

The MANOVA found that only the indicators of abstinence and tolerance had statistically significant effects on several of the dimensions of problematic social network use. Gender, on the other hand, did not show a significant multivariate effect, and no significant interactions were found between gender and addictive indicators, suggesting that the effects of abstinence and tolerance are consistent across sexes.

These findings allow us to infer that, although the female gender has previously been identified as more vulnerable to problematic use of social networks, once clear signs of addiction such as abstinence or tolerance manifest themselves, the differences between genders tend to disappear, affecting men and women in a similar way. This suggests that the problematic indicators of use (abstinence and tolerance) are highly pervasive across genders.

Finally, we would like to highlight that the correlational study allowed us to consider certain variables as relevant risk factors due to their correlation with problematic use, such as the time spent using mobile phones and social networks, the number of platforms used, which also correlated with the search for control, psychological dependence and loss of productivity.

In addition to the correlation between TikTok use and problematic use (0.162^**^), psychological dependence (0.128^*^), social recognition (0.151^**^), and the search for control (0.111^*^), these data join the latest research warning about this social network due to its high addictive potential (Qin et al., 2022) and its harmful consequences, such as memory and attention impairment (Chiossi et al., 2023), depression, and anxiety (Krentz et al., 2024).

This study has certain limitations that should be considered when interpreting the results. First, the methodological design used prevents causal relationships from being established between the variables analyzed. Second, the nature of the data obtained through self-reporting instruments, which could introduce biases inherent to this type of measurement, such as social desirability bias. Additionally, the use of non-probabilistic incidental sampling restricts the degree to which the findings can be generalized to other populations.

Despite these limitations, the study has several methodological and conceptual strengths. Among these, the large participant size (*N* = 526) stands out, which includes participants with diverse academic backgrounds, reinforcing the robustness and representativeness of the results. Likewise, the combination of objective measures—such as real-time usage recorded on devices—with participative measures (*ad hoc* questionnaires and the PSSNUS scale) provides a more comprehensive and accurate assessment of social media usage patterns. Finally, the manuscript contributes significantly to the advancement of knowledge about behavioral addictions by examining in detail fundamental constructs such as tolerance, relapse, and abstinence.

## 6 Conclusions

In conclusion, the three objectives of this study outline a growing problem that demands multidisciplinary attention. The results obtained reflect the need for prevention and intervention strategies, as well as the continuation of methodological exploration that allows for establishing causal relationships and delving deeper into the consequences and potential risk and protective factors.

This study has several limitations that should be considered when interpreting the results. First, its descriptive and cross-sectional ex post facto correlational design prevents the establishment of causal relationships. Longitudinal or experimental studies would be required to determine causality. Second, incidental non-probability snowball sampling may limit the generalizability of the results. Furthermore, data on social media and smartphone use were collected using an *ad hoc* questionnaire and other self-report instruments. This may introduce self-report biases such as social desirability bias.

## Data Availability

The data supporting the findings of this study are not publicly available due to the sensitive nature of human participant information and the terms of the informed consent. However, data may be made available by the corresponding author upon reasonable request. Requests to access the datasets should be directed to myriam.carbonell@ufv.es.
