# Supplementary material for: Social media and functional deterioration: indicators of problematic use in university students
Source: Front Psychol. 2025 Dec 19;16:1720760. doi: 10.3389/fpsyg.2025.1720760 (PMC12757270; doi:10.3389/fpsyg.2025.1720760)
Supplement: Supplementary file 1 [file Data_Sheet_1.PDF]

Herramientas Se ha guardado el 7 jul 2025 a las 07:19 Borrador Vista previa Publicar

Está utilizando la nueva Experiencia de cumplimentación de encuestas. Más información. Enviar comentarios.

TESIS_REDES SOCIALES

Puntuación de ExpertReview Bastante bien

CONSENTIMIENTO INFORMADO

Q1

Saltar a

Fin de encuesta si No, no quiero participar Se ha seleccionado

Consentimiento Informado
Usted tiene derecho a conocer el estudio al que va a ser sometido y las consecuencias que puede ocasionar. Este documento intenta explicarle todas estas cuestiones; léalo atentamente y consulte todas las dudas que le surjan, con Myriam Carbonell Colomer, doctoranda en Psicología en la Universidad Francisco de Vitoria o a través del correo electrónico myriam.carbonell@ufv.es. También puede contactar con la directora de la tesis, Elena Bernabéu, e.bernabeu.prof@ufv.es. Le recordamos que, por imperativo legal, tendrá que aceptar el consentimiento informado para poder participar en el estudio. El objetivo de este estudio consiste en evaluar el uso que hace de las Redes Sociales y valorar algunas variables psicológicas relacionadas mediante diversas pruebas, mayoritariamente escalas a las que usted debe responder. Posteriormente, si acepta el consentimiento y proporciona los datos necesarios (el correo electrónico), podrá ser seleccionado para realizar una segunda parte del estudio de manera presencial. Información básica relativa a la protección de sus datos de carácter personal: El responsable del tratamiento de sus datos es Myriam Carbonell Colomer. La finalidad del tratamiento es gestionar su participación en el proyecto “Abuso y Adicción a las redes sociales: un estudio psiconeurofisiológico e integrador” tal y como se describe en este documento. La legitimación del tratamiento es el consentimiento del interesado marcando las casillas destinadas a tal efecto. Sus datos personales no serán comunicados a terceros. Los datos serán conservados mientras sea necesario para los fines de investigación científica que persigue el proyecto de investigación anteriormente señalado y, una vez finalizado el , proyecto de investigación, los datos podrán anonimizarse debido al interés científico del estudio. Si en algún momento nos facilita datos de terceras personas, le informamos de que queda obligado a informar al interesado sobre el contenido de esta cláusula. El participante queda informado que no puede facilitar información que identifique a terceros sin obtener su consentimiento previo y por escrito. Si no acepto y autorizo a que mis datos sean tratados por Myriam Carbonell Colomer, con la finalidad de participar en el proyecto de investigación y para remitirme, por cualquier medio, incluidos los electrónicos (a modo enunciativo, pero no limitativo, correo electrónico), comunicaciones relativas a mi participación en el proyecto de investigación referenciado.
CONSECUENCIAS SEGURAS
El estudio que vamos a realizar no tiene ningún riesgo ni consecuencia para usted. Las pruebas son muy sencillas y no requieren mucho esfuerzo para realizarlas. Aceptando realizar el estudio confirmo que he leído la información que se me ha entregado. He comprendido las explicaciones que se me han facilitado, me han permitido un contacto para aclarar todas las dudas y preguntas que pueda plantearme. También comprendo que, en cualquier momento y sin necesidad de dar ninguna explicación, puedo revocar el consentimiento que ahora presto. Por ello, manifiesto que me considero satisfecho/a con la información recibida y que comprendo la indicación y las consecuencias de este estudio.

- ○ Si, quiero participar
- ○ No, no quiero participar

Importar de biblioteca

Añadir una pregunta

Datos Sociodemográficos

Q2

A continuación se le va a preguntar datos descriptivos. Recuerde que sus datos no serán públicos.

Q3

Indique su género *

- ◯ Mujer
- ◯ Hombre
- ◯ Prefiero no contestar

Q5

Indique su edad *

Q6

Indique sus estudios actuales o los últimos realizados si actualmente no está estudiando *

- ◯ Secundaría
- ◯ Bachillerato
- ◯ Grado Medio
- ◯ Grado superior
- ◯ Máster Universitario

Q8

Indique un correo de contacto si desea participar en la siguiente parte del estudio. De lo contrario ponga un 0.

Q177

GENERE EL SIGUIENTE CÓDIGO PARA QUE SUS DATOS SÉAN ANÓNIMOS
PRIMERA LETRA DEL NOMBRE + PRIMERA LETRA DEL APELLIDO + 3 ÚLTIMOS DÍGITOS DEL TELÉFONO MÓVIL
EJEMPLO: ANA LÓPEZ 634452137 = AL137

Importar de biblioteca | Añadir una pregunta

REDES SOCIALES

Q10

A continuación se procederá a preguntar en relación al consumo de Redes Sociales. Recuerde que sus datos no serán públicos.

Q12

¿A qué edad tuviste tu primer teléfono móvil con acceso a internet?

Q13

Saltar a

Fin del bloque si ¿A qué edad te creaste una ... Es igual que 0

¿A qué edad te creaste una cuenta/perfil en tu primera red social?

Importar de biblioteca

Añadir una pregunta

Datos RRSS

Q9

¿Qué red fue la primera donde se generó un perfil?

- Whatsapp
- Twitter
- Instagram
- Facebook
- TikTok
- Snapchat
- Tuenti
- Telegram
- Youtube
- Otra

Q190 *

Actualmente qué redes sociales utilizas. Puedes seleccionar todas las Redes que uses.

- ☐ Whatsapp
- ☐ Twitter
- ☐ Instagram
- ☐ Facebook
- ☐ TikTok
- ☐ Snapchat
- ☐ Tuenti
- ☐ Telegram
- ☐ Youtube
- ☐ Otra

Q15 *

¿Cuál de estas Redes Sociales tiene descargada en el teléfono móvil?

- ☐ Whatsapp
- ☐ Instagram
- ☐ TikTok
- ☐ Twitter
- ☐ Facebook
- ☐ Snapchat
- ☐ Telegram
- ☐ Otra

Q106 *

Tras haberte generado una cuenta/perfil en cualquiera de las redes sociales que tenía ¿alguna vez ha **PENSADO en quitártela? (No necesariamente debe habérsela quitado tras pensarlo)**

- ○ No
- ○ Si

Q16

Saltar a

¿En alguna ocasión has realizado algu... si No Se ha seleccionado

Tras haberte generado una cuenta/perfil en cualquiera de las redes sociales que tenía ¿alguna vez **has decidido eliminarla**?

- No
- Sí

Salto de página

Q18

¿De que red social te quitaste la cuenta/perfil? Puedes seleccionar todas aquellas que te hayas eliminado alguna vez (Cuenta tanto eliminarse el perfil como eliminar la cuenta temporalmente sin eliminar el perfil)

- Instagram
- Twitter
- Facebook
- Snapchat
- Telegram
- Whatsapp
- TikTok
- Youtube
- Otra

Q20 *

¿Cuál fue el motivo por el que decidiste quitártela? Puedes seleccionar todas las respuestas con las que te sientas identificado. EN ORDEN DE PESO, es decir en primer lugar el motivo principal por la que decidiste quitártela.

Me hacía perder mucho tiempo	1
Me hacía sentirme mal/culpable/sentimientos desagradables	2
Ya no se usaba	3
No me hacía bien psicológicamente	4
No me gustaba lo que me ofrecía	5
Para centrarme en los estudios en época de exámenes/trabajos etc	6
Otro	7

Q104 *

¿Tras desinstalar/eliminar la aplicación has sentido malestar (ansiedad, insomnio, nerviosismo etc) por no poder acceder a ella?

- Si
- No

Q21 *

Saltar a

¿En alguna ocasión has realizado algu... si No Se ha seleccionado

Después de desinstarlarla/s ¿has vuelto a abrirte una cuenta/perfil en esa MISMA red social?

- Si
- No

Q141

¿Cuánto tiempo aproximado pasó entre que se eliminó la red social y se la volvió a instalar o a meter en ella?

- <1 SEMANA
- ENTRE 1 SEMANA- 1 MES
- MÁS DE UN MÉS
- UN AÑO APROXIMADAMENTE
- MÁS DE UN AÑO

Q26

¿Cuál cree que fue el motivo por el que se volvió a meter en esa Red Social? Puede marcar todas las respuestas que desee.

- Aburrimiento
- Me sentía fuera de la sociedad
- Enterarme de lo que hacía la gente
- Para subir contenido propio
- Para distraerme
- Pasaba por un momento malo
- Otra

Q27

Saltar destino Ir al origen del salto

Saltar destino Ir al origen del salto

¿En alguna ocasión has realizado alguna conducta tras verla realizarse en una red social? Por ejemplo un challenge/reto de tiktok o beber alguna bebida alcohólica al ver gente haciéndolo, etc..?

- Si
- No

Q57 *

Selecciona todas aquellas conductas que consideras que has realizado por haber visualizado contenido en relación en sus Redes Sociales. Recuerde que sus datos nunca serán públicos.

- ☐ Beber Alcohol
- ☐ Fumar o consumir otras sustancias
- ☐ Ver pornografía
- ☐ Retos/challenges de internet
- ☐ Unirse a la crítica de alguien por redes (insultar)
- ☐ Autolesionarse
- ☐ Ejercicio
- ☐ Leer
- ☐ Juegos online (apuestas)
- ☐ Subir/pasar fotos eróticas

Q58 *

¿Te arrepentiste de haber realizado estas conductas?

- ○ Si
- ○ No

Q59 *

¿Consideras que si no hubieras visto contenido en redes sociales en relación con estas conductas no las habrías realizado en esa ocasión?

- ○ Si
- ○ No

Q48 *

¿Te gustaría disminuir el número de redes sociales que tiene? ¿o el tiempo que pasasen ella?

- ○ Si, me gustaría reducir ambas
- ○ No, no me gustaría reducir ninguna
- ○ Solo disminuiría mi tiempo de uso
- ○ Solo disminuiría el número de redes sociales que tengo

Importar de biblioteca

Añadir una pregunta

Tiempo de Uso

Q28

A continuación vas a contestar a unas preguntas en relación al tiempo de Uso de Redes Sociales

Q29 *

¿Cuánto consideras que es su tiempo de uso del teléfono móvil?

- Menos de dos horas
- 2h
- 3h
- 4h
- 5h
- Más de 5h

Q30 *

¿Cuánto de este tiempo consideras que invierte en el uso de las redes sociales?

- Menos de dos horas
- 2h
- 3h
- 4h
- 5h
- Más de 5h

Q35

Métase en Ajustes de Teléfono, en bienestar digital e indica cuál fue el tiempo de uso total del móvil que tuviste ayer indicando el día que fue.
Por ejemplo si está haciendo el cuestionario un martes debería poner: Lunes, 4h, 37m (tiempo de uso)

Q36

Dentro del bienestar digital, en tiempo de pantalla indica cuanto tiempo has gastado en cada red social el día anterior. (No cuentes Youtube ni Plataformas de Películas/Series)
Por ejemplo:
Twitter: 1,37h
Whatsapp: 0,37h
Instagram: 2,04h

Q61

¿Te consideras activo o pasivo dentro de las redes sociales?
Activo: Semanalmente subo fotos/videos, comento, mantengo conversaciones etc.
Pasivo: No suelo subir contenido, comentar etc. Prefiero ver que hacen los demás

- ○ Activo
- ○ Pasivo

Q47

¿Consideras que tu tiempo de uso en las redes sociales ha aumentado desde que te creaste un perfil ? Invirtiendo cada vez más tiempo

- ○ No
- ○ Sí

Importar de biblioteca Añadir una pregunta

TEST PSSNUS

Descripción PSSNUS

Indique el grado de acuerdo o desacuerdo que tiene actualmente con cada afirmación sobre el uso del smartphone. Cuando se habla de redes sociales (RR.SS) se hace referencia a cualquiera de ellas y cuando se habla de Whatsapp a cualquier aplicación de mensajería instantánea. No deje ninguna respuesta en blanco, por favor.

PSSNUS 1

Cuando estoy mucho rato sin mi smartphone me siento intranquilo

- TOTALMENTE EN DESACUERO
- EN DESACUERDO
- ALGO EN DESEACUERDO
- NI DE ACUERDO NI DESACUERDO
- ALGO ACUERDO
- DE ACUERDO
- TOTALMENTE DE ACUERDO

PSSNUS 2

Necesito revisar mi smartphone cada pocos minutos para ver si hay alguna notificación interesante o importante.

- TOTALMENTE EN DESACUERO
- EN DESACUERDO
- ALGO EN DESEACUERDO
- NI DE ACUERDO NI DESACUERDO
- ALGO ACUERDO
- DE ACUERDO
- TOTALMENTE DE ACUERDO

CONTROL 1

Esta es una pregunta de prueba por favor indique la opción TOTALMENTE DE ACUERDO

- TOTALMENTE EN DESACUERO
- EN DESACUERDO
- ALGO EN DESEACUERDO
- NI DE ACUERDO NI DESACUERDO
- ALGO ACUERDO
- DE ACUERDO
- TOTALMENTE DE ACUERDO

PSSNUS 3

Cuando me doy cuenta de que no tengo el smartphone cerca siento la necesidad de cogerlo.

- TOTALMENTE EN DESACUERO
- EN DESACUERDO
- ALGO EN DESEACUERDO
- NI DE ACUERDO NI DESACUERDO
- ALGO ACUERDO
- DE ACUERDO
- TOTALMENTE DE ACUERDO

PSSNUS 4

Aunque lo esté pasando bien, tiendo a consultar mi smartphone cada cierto tiempo.

- TOTALMENTE EN DESACUERO
- EN DESACUERDO
- ALGO EN DESEACUERDO
- NI DE ACUERDO NI DESACUERDO
- ALGO ACUERDO
- DE ACUERDO
- TOTALMENTE DE ACUERDO

PSSNUS 5

Si tengo que solucionar algún problema con alguien me resulta más sencillo hacerlo mediante mi smartphone que en persona.

- TOTALMENTE EN DESACUERO
- EN DESACUERDO
- ALGO EN DESEACUERDO
- NI DE ACUERDO NI DESACUERDO
- ALGO ACUERDO
- DE ACUERDO
- TOTALMENTE DE ACUERDO

PSSNUS 6

Cuando tengo que consolar o apoyar a alguien prefiero hacerlo por Whatsapp, ya que me hace sentir menos agobiado o avergonzado.

- TOTALMENTE EN DESACUERO
- EN DESACUERDO
- ALGO EN DESEACUERDO
- NI DE ACUERDO NI DESACUERDO
- ALGO ACUERDO
- DE ACUERDO
- TOTALMENTE DE ACUERDO

PSSNUS 7

Me cuesta más contar lo que me ocurre cara a cara que a través de mi smartphone.

- TOTALMENTE EN DESACUERO
- EN DESACUERDO
- ALGO EN DESEACUERDO
- NI DE ACUERDO NI DESACUERDO
- ALGO ACUERDO
- DE ACUERDO
- TOTALMENTE DE ACUERDO

PSSNUS 8

Cuando discuto, prefiero hacerlo por smartphone (Whatsapp, RR.SS...) ya que en persona no me atrevo

- TOTALMENTE EN DESACUERO
- EN DESACUERDO
- ALGO EN DESEACUERDO
- NI DE ACUERDO NI DESACUERDO
- ALGO ACUERDO
- DE ACUERDO
- TOTALMENTE DE ACUERDO

PSSNUS 9

Cuando miro las publicaciones (fotos, estados, etc.) de mis contactos suelo tener la sensación de que disfrutan y se divierten más que yo.

- TOTALMENTE EN DESACUERO
- EN DESACUERDO
- ALGO EN DESEACUERDO
- NI DE ACUERDO NI DESACUERDO
- ALGO ACUERDO
- DE ACUERDO
- TOTALMENTE DE ACUERDO

PSSNUS 10

A menudo siento envidia de la influencia y el éxito que tienen algunos de mis contactos en RR.SS.

- TOTALMENTE EN DESACUERO
- EN DESACUERDO
- ALGO EN DESEACUERDO
- NI DE ACUERDO NI DESACUERDO
- ALGO ACUERDO
- DE ACUERDO
- TOTALMENTE DE ACUERDO

PSSNUS 11

Me pone triste comprobar a través de mi smartphone y las RR.SS. que mi vida no es tan interesante como la de la mayoría de la gente.

- TOTALMENTE EN DESACUERO
- EN DESACUERDO
- ALGO EN DESEACUERDO
- NI DE ACUERDO NI DESACUERDO
- ALGO ACUERDO
- DE ACUERDO
- TOTALMENTE DE ACUERDO

PSSNUS 12

A menudo me enfado y pido explicaciones mediante mi smartphone a algún contacto que ha leído mis mensajes y no los ha contestado.

- TOTALMENTE EN DESACUERO
- EN DESACUERDO
- ALGO EN DESEACUERDO
- NI DE ACUERDO NI DESACUERDO
- ALGO ACUERDO
- DE ACUERDO
- TOTALMENTE DE ACUERDO

PSSNUS 13

Tiendo a revisar los comentarios que otros contactos hacen a mi pareja o amigos en RR.SS.

- TOTALMENTE EN DESACUERO
- EN DESACUERDO
- ALGO EN DESEACUERDO
- NI DE ACUERDO NI DESACUERDO
- ALGO ACUERDO
- DE ACUERDO
- TOTALMENTE DE ACUERDO

PSSNUS 14

Si escribo con mi smartphone a algún contacto que está "en línea" y no me contesta suelo sentirme ofendido.

- TOTALMENTE EN DESACUERO
- EN DESACUERDO
- ALGO EN DESEACUERDO
- NI DE ACUERDO NI DESACUERDO
- ALGO ACUERDO
- DE ACUERDO
- TOTALMENTE DE ACUERDO

PSSNUS 15

Con frecuencia tardo más tiempo del necesario al realizar tareas por entretenerme con mi smartphone.

- TOTALMENTE EN DESACUERO
- EN DESACUERDO
- ALGO EN DESEACUERDO
- NI DE ACUERDO NI DESACUERDO
- ALGO ACUERDO
- DE ACUERDO
- TOTALMENTE DE ACUERDO

PSSNUS 16

En ocasiones consultar mi smartphone hace que sea difícil concentrarme en las tareas que realizo.

- TOTALMENTE EN DESACUERO
- EN DESACUERDO
- ALGO EN DESEACUERDO
- NI DE ACUERDO NI DESACUERDO
- ALGO ACUERDO
- DE ACUERDO
- TOTALMENTE DE ACUERDO

PSSNUS 17

A menudo el uso de mi smartphone hace que no me ocupe de tareas que tenía pendientes.

- TOTALMENTE EN DESACUERO
- EN DESACUERDO
- ALGO EN DESEACUERDO
- NI DE ACUERDO NI DESACUERDO
- ALGO ACUERDO
- DE ACUERDO
- TOTALMENTE DE ACUERDO

PSSNUS 18

Con frecuencia me doy cuenta de que consultar mi smartphone me hace menos productivo.

- TOTALMENTE EN DESACUERO
- EN DESACUERDO
- ALGO EN DESEACUERDO
- NI DE ACUERDO NI DESACUERDO
- ALGO ACUERDO
- DE ACUERDO
- TOTALMENTE DE ACUERDO

Importar de biblioteca | Añadir una pregunta

UPPS | 21 Preguntas

OCDUS-T | 14 Preguntas

"CUESTIONARIO DE SENTIDO EN LA VIDA" (MLQ) | 11 Preguntas

DASS-21 | 22 Preguntas

EEQ bio | 11 Preguntas

AUDIT | 11 Preguntas

WLEIS | 5 Preguntas

COMUNICACIÓN INTERFAMILIAR | 11 Preguntas

Añadir bloque

Fin de encuesta

Gracias por dedicarle tiempo a esta encuesta.

Se han registrado sus respuestas.
