# Supplementary material for: Social media and functional deterioration: indicators of problematic use in university students
Source: Front Psychol. 2025 Dec 19;16:1720760. doi: 10.3389/fpsyg.2025.1720760 (PMC12757270; doi:10.3389/fpsyg.2025.1720760)
Supplement: Supplementary file 2 [file Table_1.DOCX]

Supplementary Material

# Supplementary Data

ANEXO 1

# Supplementary Figures and Tables

**Table 2**

*Usual use of different social networks by study participants*

|  | WhatsApp | Twitter | Instagram | Facebook | TikTok | Snapchat | Tuenti | Telegram |
| --- | --- | --- | --- | --- | --- | --- | --- | --- |
| Woman  (n = 362) | 360 (99.4%)** | 105 (29.0%) | 351 (97.0%) | 11 (3.0%) | 302 (83.4%)** | 60 (16.6%) | 0 (0.0%) | 20 (5.5%) |
| Men  (n = 164) | 162 (98.8%) | 76 (46.3%)* | 148 (90.2%)** | 4 (2.4%) | 88 (53.7%) | 15 (9.1%) | 0 (0.0%) | 37 (22.6%)** |
| Total  (n = 526) | 522 (99.2%) | 181 (34.4%) | 499 (94.9%) | 15 (2.9%) | 390 (74.1%) | 75 (14.3%) | 0 (0.0%) | 57 (10.8%) |

*Note:* *Values are presented as frequency (percentage). n = sample size. Significant Differences * p<*.05*; **p<*.01

**Table 5**

*Percentages of repetition of behaviour by gender*

|  | Men | Woman | | Total |
| --- | --- | --- | --- | --- |
| Drink alcohol | 24 (35.3%) | | 41 (60.3%) | 65 (12.4%) |
| Smoking or using other substances | 12 (36.4%) | | 19 (57.6%) | 31 (5.9%) |
| Watch porn | 30 (69.8%) | | 12 (27.9%) | 42 (8.0%) |
| Challenges | 62 (20.4%) | | 240 (78.9%) | 302 (57.4%) |
| Joining in criticism/insults | 24 (49.0%) | | Woman | 49 (9.3%) |
| Self-harm | 2 (18.2%) | | 41 (60.3%) | 10 (1.9%) |
| Exercise | 109 (28.9%) | | 19 (57.6%) | 375 (71.3%) |
| Read | 56 (19.5%) | | 12 (27.9%) | 286 (54.4%) |
| Online games (bets) | 42 (67.7%) | | 240 (78.9%) | 59 (11.2%) |
| Upload/pass erotic photos | 4 (33.3%) | | Woman | 12 (2.3%) |

*Note:* *Values are presented as frequency (percentage)*

**Table 7**

*Frequency of reasons for deleting an RRSS*

|  | Men | Woman | Total |
| --- | --- | --- | --- |
| It was wasting my time | 58.40% | 51.10% | 53.70% |
| Made me feel guilty/bad/unpleasant feelings | 36.60% | 35.70% | 36.20% |
| No longer in use | 27.70% | 28.50% | 28.80% |
| It didn't do me any good psychologically | 43.60% | 39.80% | 41.40% |
| I didn't like what it offered | 44.60% | 50.20% | 48.80% |
| To focus on studies/jobs | 31.70% | 32.60% | 32.80% |
| Another reason | 75.20% | 78.30% | 77.60% |

**Table 8**

*Relapse time (uninstallation. recovery of the social network)*

|  | Men | Woman | Total |
| --- | --- | --- | --- |
| <1 week | 10.20% | 19.40% | 17.40% |
| 1 week- 1 month | 40.70% | 29.40% | 32.20% |
| >1 month | 27.10% | 35.30% | 33.08% |
| Approx. 1 year | 16.90% | 8.20% | 10.40% |
| >1 year | 5.10% | 7.60% | 7.00% |

## Supplementary Figures

**
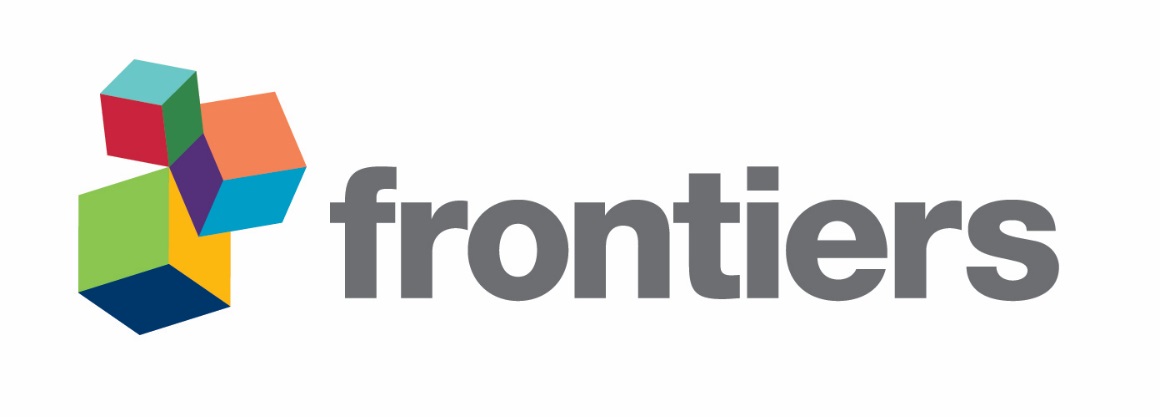
**

**Supplementary Figure 1.** The figure legends are required to have the same font as the main text. 12 point normal Times New Roman. single spaced. Please use a single paragraph for each legend and prepare the figures keeping in mind the PDF layout.
